# Genetic analysis in the bariatric clinic; impact of a *PTEN* gene mutation

**DOI:** 10.1002/mgg3.632

**Published:** 2019-05-04

**Authors:** Mellody I. Cooiman, Lotte Kleinendorst, Bert van der Zwaag, Ignace M. C. Janssen, Frits J. Berends, Mieke M. van Haelst

**Affiliations:** ^1^ Department of Bariatric Surgery Rijnstate Hospital/Vitalys Clinic Arnhem The Netherlands; ^2^ Department of Clinical Genetics Amsterdam UMC Vrije Universiteit Amsterdam Amsterdam The Netherlands; ^3^ Department of Clinical Genetics Amsterdam UMC University of Amsterdam Amsterdam The Netherlands; ^4^ Department of Genetics University Medical Centre Utrecht Utrecht The Netherlands

**Keywords:** bariatric surgery, PTEN gene, PTEN hamartoma tumor syndrome, sleeve gastrectomy

## Abstract

**Background:**

Pathogenic *PTEN* gene mutations are known to cause PTEN tumor hamartoma syndrome. Recent studies also suggest a role for *PTEN* mutations in the pathogenesis of obesity. No *PTEN* mutations have been reported among bariatric surgery patients and obesity treatment results are unknown. Since preventive screening for associated tumors is offered to patients with molecular proven PTEN hamartoma tumor syndrome, recognition of this condition in the bariatric surgery clinic is important.

**Method:**

We present a patient with morbid obesity who carries a known pathogenic *PTEN* mutation, identified at the bariatric surgery clinic using an obesity gene panel consisting of 52 obesity–associated genes. We analyzed the weight loss response during the first 3 years after Sleeve Gastrectomy.

**Results:**

At 1, 2 and 3 years after surgery, the patient achieved a Total Body Weight Loss of 39.4%, 48.8% and 44.9%, respectively. This corresponds to the results of a control group of 18 female patients with normal genetic test results.

**Conclusion:**

Our patient illustrates the importance of recognizing this serious genetic condition for which preventive cancer screening options are available. The positive weight loss results after Sleeve Gastrectomy suggest that this could be a successful treatment option for obesity patients with *PTEN* mutations.

## 1 INTRODUCTION

Bariatric surgery is an effective treatment option for obesity in the majority of patients (The GBD 2015 Obesity Collaborators et al., 2017 ; Le Roux & Heneghan, 2018). Besides following the criteria of the International Federation for the Surgery of Obesity and Metabolic Disorders (Fried et al., 2013), it is important to securely determine the obesity causing factors, to be able to select patients who are expected to benefit the most of weight loss surgery. Multiple lifestyle‐ or endocrine/hormonal‐ factors, but also a genetic cause of obesity could be of great importance for the onset of obesity. Unfortunately, sufficient knowledge about the role of underlying genetic factors and the effect of bariatric surgery in patients with genetic obesity is still lacking. We here describe a patient with a mutation in the phosphatase and tensin homologue (*PTEN*) gene, a tumor suppressor gene with a regulatory role in the cell proliferation process. Patients with PTEN hamartoma tumor syndrome (PTEN HTS) usually present with mucocutaneous lesions (90%–100%), thyroid abnormalities (50%–67%), macrocephaly (38%) or genito–urinary abnormalities (44%) in combination with a family history of different types of cancers (Dutch Guidelines, 2014; Eng, 2016). Less often diagnosis can be made in children with a combination of macrocephaly and/or mild intellectual deficit. Recent studies have also suggested a role for *PTEN* mutations in the pathogenesis of obesity (Stambolic et al., 2000). As far as we are aware, this is the first report of an obese patient with a *PTEN* mutation who successfully underwent bariatric surgery.

## 2 CASE PRESENTATION

**Figure 1:**
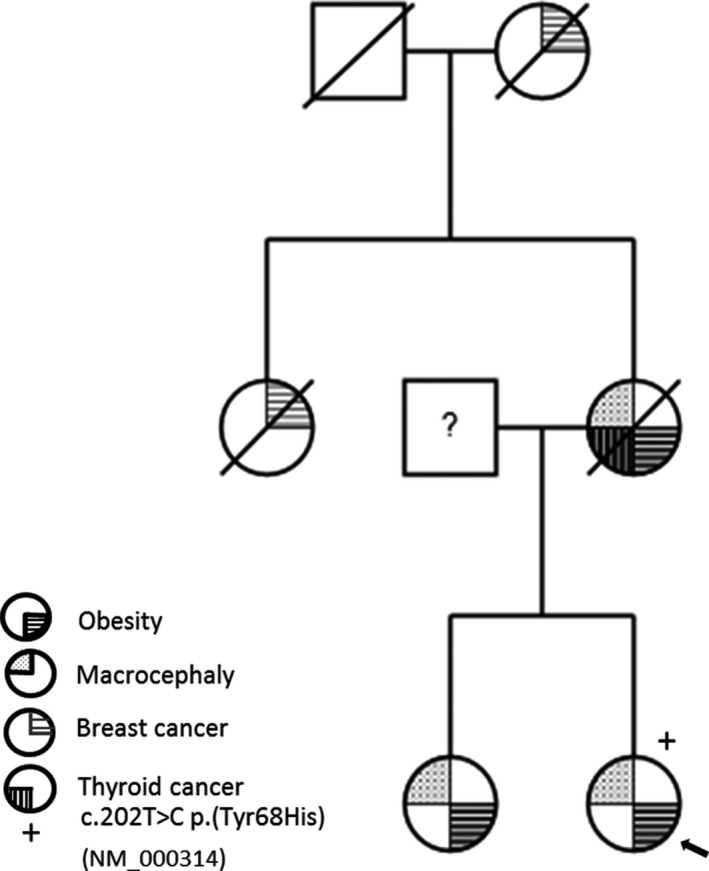
Pedigree

The index patient was a 34‐year old female referred to the bariatric clinic by the general practitioner on her own request to treat her morbid obesity. She was born with a normal birth weight but large head circumference for which she never had a diagnostic analysis. At the age of five, her body weight was already significantly higher compared to her peers. No specific life events could explain her obesity. Cognitive development was normal and she followed normal education. She underwent treatment for recurrent nasal polyps. Her mother also had a large head size and suffered from morbid obesity as well. She was diagnosed with thyroid cancer and died from a pulmonary embolism after placement of an Adjustable Gastric Band. A maternal aunt was diagnosed with breast cancer before the age of 50 and the maternal grandmother died from breast cancer at young age. The younger sister of the index patient was overweight and was reported to also have a large head size (Figure 1).

Since childhood, the index patient followed several different coaching programs to change her eating behavior and exercise pattern to induce weight loss. She lost weight several times but was never able to maintain her weight loss. At the time of the intake procedure at the bariatric clinic, her height was 1.69 m (*SD* −0.2) and weight 164 kg (*SD* +6.8), resulting in a Body Mass Index (BMI) of 57.6 kg/m^2^ and a predominant abdominal obesity. Head size was not measured at that time since this is not part of bariatric screening procedures. Biochemical analysis of the blood revealed no abnormalities, and excluded endocrine hormonal disorders such as hypothyroidism. The fasting glucose level was 5.9 mM.

The combination of early onset morbid obesity resulted in suspicion of a genetic cause of her obesity. She was offered diagnostic genetic analysis of 52 obesity–associated genes to identify a possible underlying genetic obesity cause.

The patient was eligible for bariatric surgery and underwent a sleeve gastrectomy without complications (performed in 2014 using a standardized fashion). At 1, 2 and 3 years after surgery, she achieved a percentage Total Body Weight Loss of 39.4, 48.8 and 44.9, respectively. This resulted in a current BMI of 30.1 kg/m^2^. This was within the range of the results which were observed in a control group of 18 female patients, with a negative obesity genetic test result. These female patients were matched for age and BMI and achieved a percentage Total Body Weight Loss (TBWL) of 30.3 after 1 year, 31 after 2 years and 30 after 3 years of follow‐up.

A few months after surgery, the result of the obesity gene panel analysis was returned and showed heterozygosity for a known pathogenic mutation in the *PTEN* gene (NM_000314.4): c.202T>C p.(Tyr68His). This mutation has been described previously in patients with PTEN Hamartoma Tumor Syndrome (PTEN HTS) (Marsh et al., 1998). No other pathogenic mutations were shown in the remaining 50 obesity–associated genes (Table 1). At the genetic clinic, a head circumference of 63 cm (+5*SD*) and pedigree analysis (family history of multiple tumors) further supported the molecular diagnosis of PTEN HTS.

**Table 1:** Obesity gene panel 2014

*ALMS1*	*BBS12*	*IRS4*	*MKKS*	*PCSK1*	*TBX3*
*ARL6*	*BDNF*	*KIDINS220*	*MKRN3*	*PHF6*	*THRB*
*BBS1*	*CCDC28D*	*LEP*	*MKS1*	*POMC*	*TMEM67*
*BBS2*	*CEP290*	*LEPR*	*MRAP2*	*PRKAR1A*	*TRIM32*
*BBS4*	*CRHR2*	*LZTFL1*	*NDN*	*PTEN*	*TTC8*
*BBS5*	*FLOT1*	*MAGEL2*	*NTRK2*	*SIM1*	*TUB*
*BBS7*	*G6PC*	*MC3R*	*PAX6*	*SNRPD2*	*WDPCP*
*BBS9*	*IRS1*	*MC4R*	*PTHB1*	*SNRPN*	
*BBS10*	*IRS2*	*MCHR1*	*PCK1*	*SPG11*	

Custom Agilent SureSelect target enrichment assay followed by massive parallel sequencing on SOLiD5500XL sequencer: analysis of protein coding and flanking intronic sequences of 52 obesity and obesity comorbidity associated genes.

According to the PTEN HTS guidelines, patients with pathogenic *PTEN* mutations are advised to visit the outpatient clinic for familial tumors, for lifelong surveillance of tumors that are associated with the *PTEN* mutations (Dutch Guidelines, 2014; Eng, 2016). Our patient underwent additional biochemical laboratory‐ and ultrasound screening to exclude thyroid gland carcinoma. Besides a few benign nodules on the ultrasound, no abnormalities could be determined. A yearly follow‐up ultrasound of her thyroid gland and yearly serum thyroid stimulating hormone analysis was advised. Screening for breast, endometrium and colorectal cancer, also revealed no anomalies.

## 3 DISCUSSION

Although obesity is suggested to be a multifactorial condition, mostly caused by our changing obesogenic environment, an underlying genetic defect has been reported in approximately 2%–15% of morbidly obese patients (Foucan et al., 2017; Kleinendorst et al., 2018). Mutations in the melanocortine‐4 receptor (*MC4R*) gene are the most common cause of monogenic obesity, with a prevalence of 0.5%–5.8%, with the highest values expected in cohorts with early onset obesity (Valette et al., 2012). Since genetic obesity diagnoses are often difficult to establish in obese adults, it is expected that part of the patients who undergo a bariatric surgical treatment might have an underlying genetic cause of obesity. Implementation of next generation sequencing analysis in daily clinical obesity care facilitates the identification of genetic causes of obesity. Because of the early onset morbid obesity, a genetic cause of obesity was suspected in our patient. There were no contra‐indications to perform bariatric surgery, since this was the only remaining treatment option to achieve durable weight loss.

Monogenic obesity conditions are most often detected during childhood when patients present a combination of congenital malformations, dysmorphic features and/or intellectual problems. The combination of morbid obesity and macrocephaly could also suggest a 16p11.2 deletion syndrome. The prevalence of this genetic condition in the general population is estimated at 3 in 10,000. It is mostly associated with autism spectrum disorder and learning‐ and speech problems, but it is also a 43‐fold increased risk for morbid obesity (Maillard et al., 2016; Walters et al., 2010). The family history of breast‐ and thyroid cancer and the normal development in our patient made this diagnosis less likely.

The association of obesity and *PTEN* mutations is not well understood. Garcia‐Cao et al. (2012) and Ortega‐Molina et al. (2012) showed that overexpression of *PTEN* in mice leads to reduced body weight and size, combined with hyperphagia. This suggested a poor energy storage capacity, which was confirmed by calorimetric measurements showing increased energy expenditure and oxygen consumption in these mice (Ortega‐Molina & Serrano, 2013). This was further supported by the finding of elevated activity of brown adipose tissue in *PTEN* overexpressed mice (Garcia‐Cao et al., 2012).

In humans, Pal et al. (2012) showed a strong association between *PTEN* loss of function mutations resulting in expected haploinsufficiency and the presence of obesity. Fifteen *PTEN* mutation carriers had a mean BMI of 32 kg/m^2^ (range 23–42) compared with 26 kg/m^2^ in fifteen matched controls (range 15–48), showing that the *PTEN* affected patients were clinically significantly overweight (*p* = 0.001). Data from bone densitometry, did however show no significant differences in lean mass, bone mineral content or total fat between the patients with a *PTEN* mutation and controls. The authors state that the higher BMI in patients with *PTEN* mutation could be attributable to an increase in adipose tissue. Their presented data do however not yet support this conclusion, since there was no significant difference in skinfold thickness between the patients and the controls (Leow, 2012; Ortega‐Molina et al., 2012; Pal et al., 2012). So the exact role of *PTEN* associated obesity still remains unclear and further research is needed to determine the mechanism behind the reported higher BMI in patients with *PTEN* mutation.

PTEN HTS is rare and difficult to diagnose if not familiar to the clinician. Especially since the prevalence in selected groups, such as obese patients in the bariatric clinic, is not known.

The results after sleeve gastrectomy were good in our patient and comparable with a control group of matched patients. However, no definitive conclusion can be drawn from this positive result since this is the first report of a patient with a *PTEN* mutation who underwent bariatric surgery. More research is needed to determine the best treatment possibilities for these patients.

Although weight loss reduces the risk of cancer development in the general population, timely identification of *PTEN* mutations in early onset obesity patients can further result in a major health benefit. This is also of great importance for other family members who are at risk of sharing the same genetic defect. Since the mother, the maternal aunt and maternal grandmother were reported to have clinical features fitting with a diagnosis of PTEN HTS, it is highly suggestive that our patient inherited a familial *PTEN* mutation. The sister of our patient was referred to the genetic department as well. Unfortunately we do not have any further information on her.

In conclusion, we here report a case with morbid obesity associated with a pathogenic *PTEN* mutation. The sleeve gastrectomy in this case resulted in successful weight loss in the first 3 years after surgery, but more cases with a *PTEN* mutation who underwent bariatric surgery need to be reported. Long term follow‐up results and further clarification of *PTEN* mutations in the pathogenesis of obesity, might lead to further personalized treatment options.

## CONFLICT OF INTEREST

The authors declare no conflict of interest.
